# Incidence of neonatal venous thromboembolism: a systematic review and meta-analysis of the literature

**DOI:** 10.1016/j.rpth.2026.106630

**Published:** 2026-05-08

**Authors:** Marie-Claude Pelland-Marcotte, Norma Maria Herrera Perez, Elisabeth Boileau, Eve Bouhêlier, Heleen van Ommen, Rukhmi Bhat

**Affiliations:** 1 Department of Pediatrics, CHU de Québec—Centre Mère-Enfant Soleil, Quebec City, Quebec, Canada; 2 Research Center of the CHU de Québec, Axe Reproduction, Santé de la Mère et de l’Enfant, Quebec City, Quebec, Canada; 3 Centre de recherche du CHU de Québec, Quebec City, Quebec, Canada; 4 Faculty of Medicine, Université Laval, Quebec City, Quebec, Canada; 5 Pediatric Hematology/Oncology, Erasmus MC Sophia Children’s Hospital, Rotterdam, The Netherlands; 6 Department of Pediatrics, Northwestern University Feinberg School of Medicine, Ann & Robert H. Lurie Children’s Hospital of Chicago, Chicago, Illinois, USA

**Keywords:** central venous catheters, epidemiology, neonates, thrombosis

## Abstract

**Background:**

Venous thrombosis is increasingly reported in neonates, but contemporary estimates of its incidence are missing.

**Objectives:**

To determine the incidence of venous thromboembolism in neonates.

**Methods:**

A literature search was performed for peer-reviewed publications (1990-2024). We included peer-reviewed studies describing incidence of venous thrombosis in neonates up to 44 weeks of corrected gestational age. Risk of bias in included studies was assessed using the “risk of bias in nonrandomized studies—of interventions” tool. We performed a meta-analysis for all included studies and stratified by comorbidities, thrombosis location, study design, and use of thrombosis screening. Publication bias was assessed using funnel plot and regression test for funnel plot asymmetry.

**Results:**

The literature search retrieved 10,862 studies, of which 82 were included in this review (*N* = 3,461,607 neonates). Most studies (98%) included neonates admitted to the neonatal intensive care unit, and several studies focused further on neonates with additional comorbidities such as congenital heart disease or prematurity. Overall, the cumulative incidence of venous thrombosis was 10.6% (95% CI, 7.5%-13.7%). Reported incidences of thrombosis were higher in studies focusing on neonates with cardiac disease and on catheter-related and portal thrombosis. Prospective studies and studies using surveillance imaging also reported higher rates of thrombosis.

**Conclusion:**

Venous thrombosis is frequent among neonates hospitalized in neonatal intensive care units, but its frequency appears to be highly variable depending on the population studied, whether clinically unsuspected thromboses are considered, and study design. Future work is urgently needed to prevent thrombosis in this population.

## Essentials

• Within the pediatric population, neonates are particularly at risk of thrombosis.
• We performed a systematic review and meta-analysis to determine the incidence of venous thromboembolism in neonates.
• The cumulative incidence of venous thrombosis was 10.6% (95% CI, 7.5%-13.7%).
• The frequency was highly variable depending on the population studied, whether clinically unsuspected thromboses were considered, and study design.

## 1 Introduction

The incidence of venous thrombosis is rising in children [1,2]. Neonates are particularly at risk of thrombosis within the pediatric population [3]. In neonates, ∼90% of thrombosis are related to a central venous catheter (CVC) [4,5]. CVCs are life-saving medical devices that allow for intravenous administration of medication, nutrition, and blood sampling. However, CVCs may cause thrombosis due to damage to the endothelial cells, introduction of a thrombogenic surface, and disruption of normal blood flow, especially in small blood vessels [6].

Neonates have a unique coagulation system characterized by decreased levels of several coagulation proteins, such as factor (F)II, FVII, FIX, FX (vitamin K-dependent proteins), FXI, and FXII (contact pathway proteins), resulting in lower thrombin generation potential, balanced with reduced levels of several natural anticoagulants, including antithrombin, protein C, and protein S, as well as reduced fibrinolytic activity. These age-related differences are magnified in premature neonates, although maturation of the coagulation system appears to be accelerated following premature birth. While this process, termed developmental hemostasis, is well-adapted in most neonates, it can precipitate bleeding and thrombotic events following other physiological stressors [[6], [7], [8]].

Venous thrombosis can lead to serious complications, such as organ dysfunction (eg, systemic hypertension and renal dysfunction following renal thrombosis, hepatic lobe atrophy, and portal hypertension following portal vein thrombosis), postthrombotic syndrome, and rarely mortality [[9], [10], [11], [12]]. Treatment of thrombosis with antithrombotic treatment may complicate the management of underlying illness and lead to systemic and/or intracranial bleeding, especially in premature infants [13].

Several prospective and retrospective studies have investigated the epidemiology of neonatal venous thrombosis; however, many questions remain unanswered. Reported incidences varied widely, ranging between 0.2% and 75% [2,[14], [15], [16], [17]], which may be influenced by the population studied, the type of neonatal intensive care unit (NICU) and level of acuity of the study site, the definition of VTE (eg, whether clinically unsuspected thromboses are considered), and whether active surveillance is performed to detect thrombosis, among other factors. In critically ill neonates, attribution of clinical signs to venous thrombosis can be especially challenging [2]. For instance, umbilical vein catheters are commonly used in this population and may lead to portal vein thrombosis, which may easily go undetected by clinical evaluation.

Understanding the epidemiology of neonatal venous thrombosis, both overall and CVC-related, is important as it quantifies the burden of disease and identifies subpopulations at particularly high risk who could benefit from preventative measures such as having policies or procedures in place for early central catheter removal. Thus, we performed a systematic review and meta-analysis of the literature to synthesize the incidence of venous thrombosis in neonates, incorporating data from prospective and retrospective cohort studies, case–control studies, and cross-sectional studies.

## 2 Methods

The systematic review and meta-analysis abided by the Preferred Reporting Program for Systematic Reviews and Meta-Analyses (PRISMA) statement [18]. The protocol for this review was recorded on the PROSPERO registry (registration number: CRD42024518801).

### 2.1 Data sources and search

A professional librarian performed a search in the Medline and Embase databases on August 7, 2024. All databases were searched from 1990 to present, with the search not limited by language. The titles of articles listed in the reference sections of the included articles were screened to identify other potentially relevant publications or relevant gray literature. Supplementary Table 1 shows the search strategy used in Medline.

### 2.2 Study selection

We included randomized and nonrandomized trials, retrospective and prospective cohort studies, case–control studies, or case series (≥10 patients) published in peer-reviewed journals. Studies were included if at least 90% of the included population were neonates, defined as full-term infants ≤28 days of age or preterm infants with corrected gestational age ≤44 weeks, and if they reported the incidence of venous thrombosis, including both symptomatic and clinically unsuspected events.

Titles and abstracts of studies retrieved by the search strategy and full text of potentially eligible studies were screened independently by 2 of 4 investigators (articles were divided equally among M.-C.P.-M., N.P., E. Boileau, and R.B., so that individual articles were assessed independently by 2 researchers). Any disagreement over the eligibility of studies was resolved through consensus. The web-based software Covidence (Veritas Health Innovation, Melbourne, Australia; www.covidence.org) was used to streamline study selection.

### 2.3 Data extraction and quality assessment

Data extraction was conducted independently by 2 of 4 investigators (M.-C.P.-M., N.P., E. Boileau, and R.B.) using an Excel-based standardized form. Extracted information included study setting, design, sample size, patients’ characteristics, outcome definition and use of surveillance imaging, incidence, and any subanalysis performed. If important information for study eligibility or data abstraction was missing, the corresponding author was contacted via e-mail up to 3 times to obtain missing data.

Each included study was evaluated independently at the study level by 2 members of the review team for the risk of bias using the “risk of bias in nonrandomized studies—of interventions” (ROBINS-I) tool [19,20]. The risk of bias was classified as high, low, or unclear for each of the following domains: confounding, selection of participants, classification of exposure/risk factors, deviations from intended procedures, missing data, measurement of outcomes, and selective reporting.

### 2.4 Data synthesis and analysis methods

Data were presented descriptively, using median and IQR for continuous data and proportions for categorical data. Means and SDs were extracted whenever medians were not available. The incidence of venous thrombosis was reported separately for all venous thrombosis and CVC-related venous thrombosis, when applicable.

We calculated the incidence of neonatal venous thrombosis using the restricted maximum-likelihood method, with a random-effect model accounting for the methodological and clinical heterogeneity of included studies. Analyses were performed (a) for the entire cohort, (b) excluding population-based studies to capture incidence rates among hospitalized neonates, and (c) stratified by underlying comorbidity, thrombosis location, and use of screening for venous thrombosis. Additionally, we had planned a priori to perform stratified analysis based on thrombosis definition (symptomatic vs clinically unsuspected VTE), as clinically unsuspected venous thromboses appear to have a less severe clinical course in older children [21]. For the primary analysis, it was decided to discard studies with patients undergoing extracorporeal membrane oxygenation or exchange transfusion, due to the very high rate of thrombotic complications in this population; results of these studies are presented descriptively. As few studies reported the incidence of thrombosis by catheter-day, we did not perform quantitative analysis with the CVC as the unit of analysis.

Interstudy heterogeneity was quantified using the *τ*^2^ (absolute measure of between-study variance in a random effects meta-analysis) and the *I*^2^ statistic (proportion of total variation across studies due to heterogeneity) [22], where an *I*^2^ value of >50% indicated substantial heterogeneity [23]. Publication bias was assessed using funnel plot and regression test for funnel plot asymmetry. In the presence of significant funnel plot asymmetry, exploratory analyses comparing fixed and random-effect meta-analyses allowed us to determine whether asymmetry could be attributed to small-study effect. Population-based studies, given their very large sample size, were excluded from fixed-effect analysis.

Given the substantial changes in neonatal care, notably the increasing use of unfractionated heparin for CVC patency at the turn of the early 2000s, we performed a sensitivity analysis where studies published before 2000 were removed. All analyses were performed using the metafor package in R software (version 4.1, retrieved from https://cran.r-project.org) and Jamovi (version 2.6, retrieved from https://www.jamovi.org). A 2-sided *P* value of <.05 was considered statistically significant.

## 3 Results

**Figure 1:**
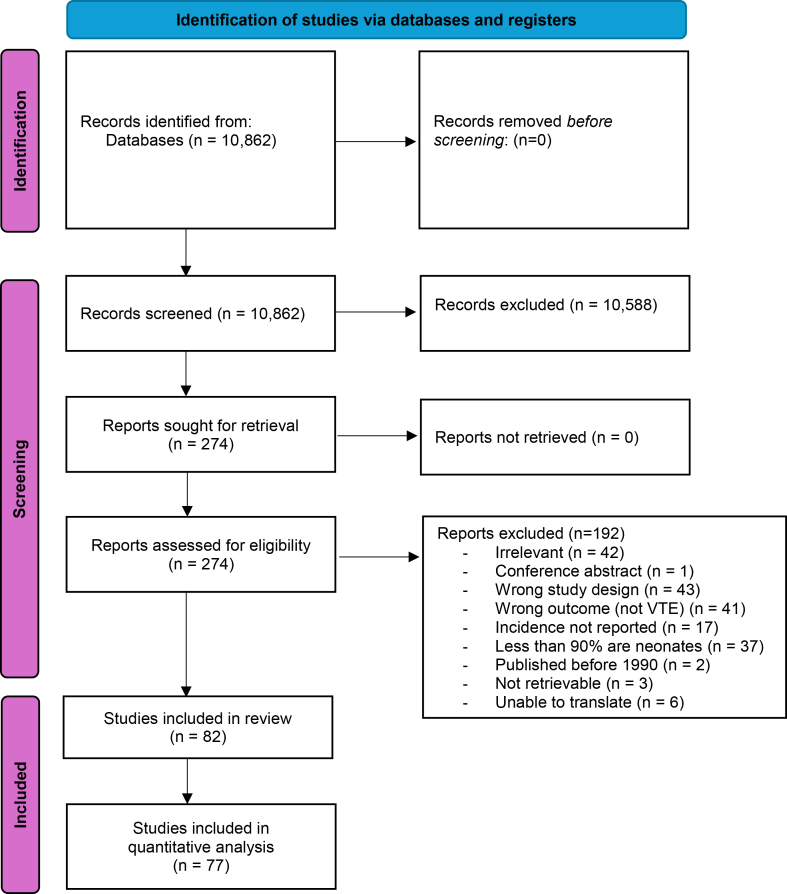
Flow chart of included studies.

The search strategy produced 10,862 unique references, of which 274 full-text articles were retrieved (Figure 1). Eighty-two studies (totaling 3,461,607 neonates) were included [5,7,16,17,[24], [25], [26], [27], [28], [29], [30], [31], [32], [33], [34], [35], [36], [37], [38], [39], [40], [41], [42], [43], [44], [45], [46], [47], [48], [49], [50], [51], [52], [53], [54], [55], [56], [57], [58], [59], [60], [61], [62], [63], [64], [65], [66], [67], [68], [69], [70], [71], [72], [73], [74], [75], [76], [77], [78], [79], [80], [81], [82], [83], [84], [85], [86], [87], [88], [89], [90], [91], [92], [93], [94], [95], [96], [97], [98], [99], [100], [101]]. Table 1 describes the characteristics of included studies. Most studies were retrospective cohort studies (*n* = 40, 49%) or prospective cohort studies (*n* = 25, 30%). While the majority of studies (*n* = 48, 59%) focused on general NICU populations, several reports focused on specific populations such as neonates with cardiac diseases (*n* = 11, 13%), premature newborns (*n* = 8, 10%), or neonates with congenital anomalies/requiring major surgeries (*n* = 5, 5%), such as intestinal malrotation or gastroschisis. Two studies used population-based registries or surveys; thus, study inclusion was not conditional on admission to the hospital or the NICU [30,67]. One study also estimated the incidence of neonatal thrombosis based on population-based estimates of live births [95]. Several reports (*n* = 34, 41%) did not mention the definition of venous thrombosis, and an additional 36 (44%) studies did not report symptomatic and clinically unsuspected venous thrombosis separately.

**Table 1:** Characteristics of included studies.

Characteristics	*N* = 82
Study design	
Case–control	10 (12)
Prospective cohort	25 (30)
Retrospective cohort	40 (49)
Randomized controlled trial	4 (5)
Other	3 (4)
Year of publication	
1990-1999	6 (7)
2000-2009	14 (17)
2010-2019	26 (32)
2020 to current	36 (44)
Geographical area	
North America	38 (46)
Europe	22 (27)
Asia	17 (21)
Other	5 (6)
Underlying conditions/clinical setting	
Population-based	2 (2)
General NICU population	48 (59)
Cardiac disease	11 (13)
Congenital anomaly	5 (6)
Critically ill infants	2 (2)
ECMO or cardiopulmonary bypass	3 (4)
Prematurity/low birth weight	8 (10)
Term or near-term infants	3 (4)
Routine imaging for VTE performed?	
Yes	30 (36)
Variable	2 (2)
No	50 (61)
Use of heparin for line patency	
Yes	26 (32)
No	20 (24)
Tested as part of the study	3 (4)
Not reported/not applicable	33 (40)

Values are presented as *n* (%).

ECMO, extracorporeal membrane oxygenation; NICU, neonatal intensive care unit; VTE, venous thromboembolism.

Surveillance imaging was performed in less than half of the studies (*n* = 32, 39%). Among studies that performed surveillance imaging, the modalities used were Doppler ultrasound (*n* = 17, 53%), echocardiography (*n* = 9, 28%), magnetic resonance with venography (*n* = 2, 6%), and a combination of echocardiography and Doppler ultrasonography (*n* = 2, 6%) or echocardiography with venography (*n* = 1, 3%). The modality was not specified in 1 study (3%). The number of surveillance imaging was highly variable, ranging from 1 to 9, as well as the timing of the planned surveillance imaging.

The risk of bias is presented in Supplementary Table 2. Most studies (*n* = 67, 82%) had a serious risk of bias in at least 1 domain, most commonly, confounding bias or risk of bias in outcome measurement, for example, when screening or diagnostic procedures for thrombosis detection were not standardized or differed between groups.

**Figure 2:**
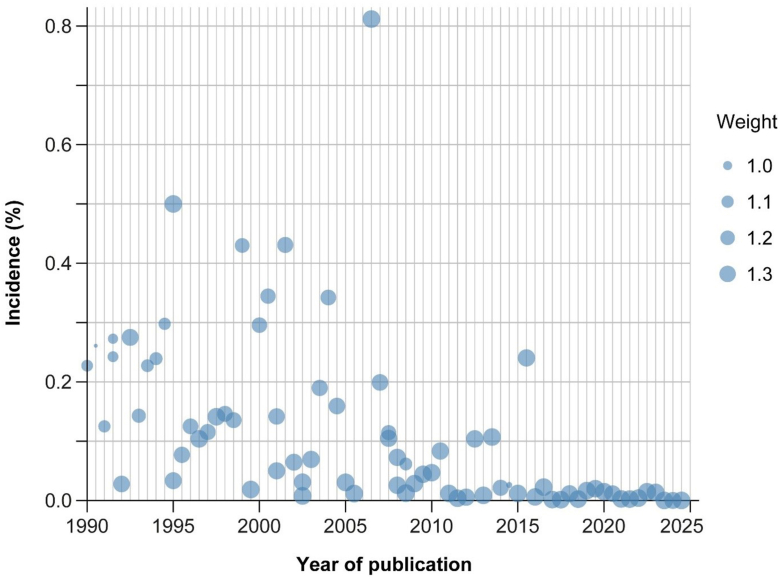
Bubble plot of the incidence of neonatal venous thromboembolism, by year of publication. Each study is represented by a bubble, with size proportional to its weight in the random-effects meta-analysis.

**Figure 3:**
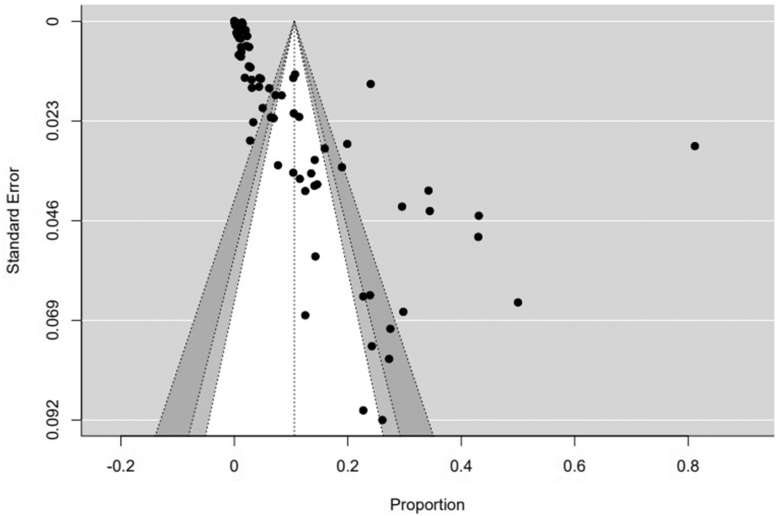
Colored-enhanced funnel plot—incidence of venous thrombosis in neonates. Regression test for funnel plot asymmetry: *Z* = 8.065; *P* < .001. The white, light gray, and dark gray areas represent the expected distribution of study results in the absence of publication bias and/or small-study effect for the following significance levels: *α* = 0.10, *α* = 0.05, and *α* = 0.01 levels, respectively.

The incidence of neonatal venous thrombosis was reported in 77 studies [5,16,17,[24], [25], [26], [27], [28], [29], [30], [31], [32], [33], [34], [35], [36], [37], [38], [39], [40], [41], [42], [43], [44], [45], [46], [47], [48], [49], [50], [51], [52], [53], [54], [55], [56], [57], [58], [59], [60], [61], [62], [63], [64], [65], [66], [67], [68],[70], [71], [72], [73], [74], [75], [76], [77], [78],[80], [81], [82], [83], [84], [85], [86], [87],^89^,[91], [92], [93], [94], [95], [96], [97], [98], [99], [100], [101]] A total of 5,345,988 patients was included in this analysis, exceeding the overall number of patients included in the systematic review (*N* = 3,461,607) as 9 studies used complementary administrative data (eg, the number of NICU admissions during the study period), totaling 1,844,381 neonates, to calculate the incidence of thrombosis, in addition to their reported sample size [5,26,27,30,35,95,97,99,101]. In the primary analysis, the pooled cumulative incidence of venous thrombosis was 10.6% (95% CI, 7.5%-13.7%) (Figure 2) and 11.0% (95% CI, 7.8%-14.2%) among studies limited to hospitalized neonates (Table 2). On the funnel plot, statistically significant asymmetry was present (regression analysis for asymmetry: *P* < .001) (Figure 3). Exploration of the asymmetry with fixed-effect model showed important differences with the original model (fixed effect: 0.84%; 95% CI, 0.80%-0.90%), suggesting small-study effect.

**Table 2:** Pooled cumulative incidence of neonatal venous thrombosis.^a^

Population	No. of studies	Pooled cumulative incidence^b^ (%)	95% CI (%)	Heterogeneity	
				τ^2^	*I*^2^ (%)
Overall	77	10.6	7.5-13.7	0.0184	100
Studies of hospitalized neonates	74	11.0	7.8-14.2	0.0188	100
Stratified by comorbidity					
Cardiac disease	10	16.5	9.9-23.1	0.0096	93
Congenital anomaly	5	5.3	0.0-10.5	0.0031	97
Prematurity	8	11.6	2.5-20.6	0.0164	99
Term or near-term	3	4.6	0.0-9.8	0.0015	75
Stratified by thrombosis location					
Catheter-related	46	9.9	6.7-13.1	0.0111	100
Intracardiac^c^	—	—	—	—	—
Portal	6	22.0	7.4-36.6	0.0311	98
Renal^c^	—	—	—	—	—
Cerebral	3	10.5	0.0-23.9	0.0124	97
Stratified by VTE screening					
Yes	28	14.5	9.6-19.4	0.0163	99
No	48	8.4	4.4-12.3	0.0189	100
Stratified by study design					
Prospective^d^	24	17.6	11.9-23.2	0.0179	99
Retrospective	37	9.2	4.4-13.9	0.0212	100

a Pooled incidence were estimated using the restricted maximum-likelihood method, with random-effect model.

b Presented in percentage for ease of reading.

c Insufficient number of studies to perform analysis.

d Includes observational prospective studies and randomized controlled trials.

Pooled estimates of the cumulated incidence of venous thrombosis in stratified analyses in presented in Table 2. Overall, venous thrombosis incidence was reported to be higher in (a) studies including neonates with cardiac disease, compared with other comorbidities; (b) studies focusing on portal and CVC-related thromboses; (c) studies using thrombosis screening; and (d) prospective studies, compared with retrospective studies.

The sensitivity analysis, which included studies published from 2000 onward, yielded similar results to the main analysis (*n* = 71 studies; pooled cumulative incidence: 10.6%; 95% CI, 7.3%-13.9%; *I*^2^ = 100%). Four studies [65,69,79,90] examined neonates undergoing extracorporeal circulation, extracorporeal membrane oxygenation, or exchange transfusion (*n* = 265 neonates). The results of these studies are summarized in Table 3.

**Table 3:** Incidence of venous thrombosis in neonates undergoing extracorporeal circulation.

Reference	Study design	Population (sample size)	Thrombosis definition	Screening	Incidence
Navaratnam et al. ^[^^67^^]^^a^	RCS	Neonates undergoing cardiac surgery under CPB (*n* = 86)	Any radiologically proven thrombus (including shunt or circuit thrombosis)	None reported	22/165 (13.3%)
Patregnani et al. ^[^^69^^]^^a^	RCS	Neonates undergoing systemic-to-pulmonary shunt placement (*n* = 75)	Shunt thrombosis	None reported	On CPB: 41/66 (62%) Off CPB: 9/9 (100%)
Sakha et al. ^[^^79^^]^^a^	PCS	Neonates undergoing exchange transfusion (*n* = 50)	Radiologically confirmed thrombus in catheter territory	Doppler ultrasound 1-2 wk following catheter removal	17/50 (34%)
Stewart et al. ^[^^90^^]^^a^	RCS	Neonates with congenital diaphragmatic hernia undergoing ECMO (*n* = 54)	Not reported	None reported	8/54 (15%)

CPB, cardiopulmonary bypass; ECMO, extracorporeal membrane oxygenation; PCS, prospective cohort study; RCS, retrospective cohort study.

a Statistically significant results at a *P* value of <.05.

### 3 Discussion

We report on the largest meta-analysis pertaining to incidence rates in neonatal thrombosis. The pooled cumulative incidence of venous thrombosis was 10.6%. The number of studies exploring thrombosis incidence has increased in the recent past, in parallel with the reported increase in venous thrombosis among critically ill pediatric patients [1,102]. Similarly, in this systematic review of 82 studies, most studies included neonates who were admitted to the NICU. Stratified analyses revealed that VTE incidence was higher among studies in neonates with cardiac disease, as well as in prospective studies or those performing active surveillance imaging.

In a systematic review of 18 studies by Song et al., the incidence of venous thrombosis was 2% (95% CI: 1%-2%) in neonates and children admitted to the intensive care unit [103]. The heterogeneity within our systematic review and in comparison with other meta-analyses is likely to be explained by differences in patient populations, outcome definition, and methodological factors. While we aimed to delve into these differences through stratified analyses, additional known and unknown variables probably impact the epidemiology of neonatal thrombosis, amid the diversity and complexity of neonatal intensive care. Further understanding of the epidemiology of neonatal thrombosis could enable patient-centered use of preventative measures in neonates at high risk, for example, targeted thromboprophylaxis or limiting prolonged CVC placement.

Several authors have recently synthesized the published evidence regarding CVC-related thrombosis in pediatric patients, confirming that thrombosis is a frequent complication of CVC. Chen et al. [104] have also reported a pooled incidence of 2% among 24 studies enrolling neonates with a peripherally inserted central catheter line insertion before 28 days of age. Finally, the pooled cumulative incidence of catheter-related thrombosis was 9.7% in a meta-analysis of 47 studies of hospitalized pediatric patients [105], similar to our pooled cumulative incidence of 9.9%.

In terms of populations, some studies focused on selected neonates with specific diseases, such as complex congenital heart disease or congenital diaphragmatic hernia. The high incidence of venous thrombosis in neonates with cardiac disease aligns with previous reports, as these infants typically accumulate several prothrombotic risk factors: requirement for CVCs and surgeries, venous stasis, and inflammatory states [[106], [107], [108]]. Of note, no data were available to describe the incidence of thrombosis in several patient groups admitted to the NICU, notably with other congenital malformations.

As expected, stratified analyses showed higher incidences of thrombosis in studies performing surveillance imaging. Surveillance for thrombotic events is not the current standard of care. The ideal surveillance protocol and its clinical relevance remain largely debated. Venography is considered the gold standard for venous thrombosis diagnosis but is rarely feasible in neonates and requires adequate peripheral venous access, sedation, and radiation [109]. In a study of 90 children with congenital heart disease, venography missed several thrombi, with a sensitivity of 36%, compared with ultrasonography [110]. In this study, ultrasonography demonstrated good sensitivity for detecting jugular and subclavian thrombi but had limited sensitivity for intrathoracic vessels, where compressibility cannot be reliably assessed. Echocardiography also appears to have a limited role in the diagnosis of venous thrombosis [77,110], although a secondary analysis of the Prophylactic Antithrombin Replacement in Kids with Acute Lymphoblastic Leukemia Treated with Asparaginase (PARKAA) study found that echocardiography detected right atrial thrombi missed by venography [111]. While a combination of tests may be required to detect neonatal thrombosis, performing multiple tests is impractical for surveillance purposes alone.

Similarly, the ideal surveillance frequency is unknown, as some thrombi might resolve spontaneously. For example, Kim et al. [56] found clinically unsuspected portal vein thrombosis in 43% of neonates with an umbilical vein catheter, but 56% of these thrombi resolved spontaneously after an average of 10 days. Similarly, Cabannes et al. [32] showed that left portal vein thrombosis developed within 10 days of umbilical vein placement in 27% of neonates, yet it spontaneously resolved in 95% of them. Altogether, this suggests that surveillance serial ultrasonography, especially if done shortly after line placement, may lead to overdiagnosis and overtreatment of clinically unsuspected portal vein thrombosis. Therefore, no current data or guideline support the use of routine venous thrombosis surveillance in clinical settings, but further research is needed to understand the short- and long-term natural history of clinically unsuspected neonatal venous thrombosis in other locations, for example, renal vein and inferior vena cava.

We had planned a priori to calculate the pooled cumulative incidence separately for symptomatic and clinically unsuspected venous thrombosis; however, this was ultimately not feasible as (a) the definition of this outcome was missing in almost half of the eligible/included studies and (b) many studies relied on administrative data and discharge diagnoses to establish the presence of venous thrombosis, thereby limiting our ability to determine whether symptoms were present. However, while clinically unsuspected venous thrombosis appears to have a less severe clinical course in children [13,21], this assumption may be problematic in critically ill, nonverbal neonates due to inherent challenges in detecting clinical signs and symptoms of venous thrombosis. Furthermore, physical examination has been previously shown to have poor accuracy in detecting deep venous thrombosis in older critically ill children [112].

The strengths of our systematic review include the thoroughness and rigor of our search and methodology, and strict adherence to methodological and reporting guidelines for systematic reviews. As a result, it represents the largest and most comprehensive review of incidence of neonatal thrombosis of available literature to date.

However, several limitations merit discussion. First, most of the included studies were retrospective reports and had a serious risk of bias, thereby limiting the robustness to determine the “true” incidence of neonatal thrombosis. Second, while we aimed to perform stratified analyses based on thrombosis location, classifying the thrombosis location was often difficult. For example, thrombi following umbilical vein catheterization could be classified as “portal” or “line-related” by the investigator, and several studies did not report thrombi location. Future studies should report separately thromboses separately based on their anatomic location and specify whether they were symptomatic or clinically unsuspected. Third, the funnel plot revealed asymmetry, suggesting the presence of small-study effect, where results tend to be more pronounced in smaller studies than in large ones. The small-study effect may be attributable to publication bias, in which studies with negative findings are less likely to be published or published in less visible journals. While publication bias is hard to confirm, it could lead to an overestimation of the “true” incidence of venous thrombosis in neonates. We aimed to minimize this bias by searching multiple databases, not limiting our search to English-language journals, and examining reference lists of included articles and relevant trial registries [113]. It is also highly plausible that small-study effect is explained by methodological differences. Prospective studies with active screening are inherently smaller than studies using large administrative databases, due to cost and feasibility constraints, but may have a higher capacity to detect thrombi. Finally, small-study effect could also reflect true heterogeneity within the neonatal population. Studies with stringent inclusion criteria focusing on severely ill neonates (eg, extreme prematurity and congenital diaphragmatic hernia) may report a higher incidence of thrombosis due to increased baseline risk. Despite these limitations, our study represents the largest and most comprehensive meta-analysis on incidence rates of neonatal thrombosis to date.

As we faced (a) high rates of clinical and methodological heterogeneity among included studies and (b) missing data regarding key variables, we propose that, for clinical research pertaining to the epidemiology of neonatal and pediatric thrombosis, researchers explicitly state key patient-, thrombus-, catheter-, and imaging-related elements (Table 4). These principles are aligned with recent recommendations for standardized definitions and future research by the International Society on Thrombosis and Haemostasis (ISTH) Scientific and Standardization Committee subcommittee on pediatric and neonatal thrombosis and can improve the quality and transparency of epidemiological research [114,115]. We also highlighted important directions for future research (Table 5).

**Table 4:** Recommended data elements for reporting of studies on the incidence of neonatal and pediatric venous thrombosis.

Domains	Data elements
Patient-related elements	• Age group (eg, gestational age) • Inpatient setting—intensive care or general ward • Underlying medical conditions • Use of thromboprophylaxis, when applicable
Thrombus-related elements	• Definition of venous thrombosis (eg, discharge code or radiologically proven) • Symptomatic vs clinically unsuspected ○ Symptomatic: CVC malfunction, defined as the inability to either draw or infuse, or requirement of tissue plasminogen activator at least once for catheter patency, should be considered a clinical sign of CVC-related thrombosis ○ Clinically unsuspected: detected by surveillance imaging vs incidental detection during imaging performed for unrelated pathology • CVC-related or non–CVC-related ○ If CVC-related: ▪ CVC type, location, and duration ▪ Use of unfractionated heparin for CVC line patency • Anatomic location
Imaging-modality related elements	• Imaging modality • Reason for imaging (eg, surveillance, non–VTE-related clinical issues, CVC dysfunction, and monitoring while on anticoagulant therapy) • Timing of surveillance, when applicable • Standardized protocol used

CVC, central venous catheter.

**Table 5:** Proposed directions for future research in neonatal venous thrombosis.

Priority areas for future research
• Prospective studies, using standardized diagnostic and reporting protocols • Diagnostic and surveillance strategies o Optimal timing of imaging o Natural history and clinical relevance of clinically unsuspected thrombosis • High-risk populations o Neonates with congenital heart anomalies o Neonates with noncardiac congenital anomalies o Surgical conditions (eg, necrotizing enterocolitis) • Long-term outcomes o Postthrombotic syndrome o Organ-specific long-term sequelae • Preventative interventions o Early removal protocols for central venous catheters o Comparison of various types of central venous catheters

## 5 Conclusion

This meta-analysis is the most comprehensive synthesis to date of the incidence of neonatal thrombosis. In our systematic review, the pooled cumulative incidence of venous thrombosis was 10.6% among neonates. Our results are important in highlighting the increasing burden of venous thrombosis in neonates, particularly among critically ill neonates, those with cardiac disease, and/or central venous catheters. Reporting of studies pertaining to neonatal and pediatric venous thrombosis should include transparent outcome definitions, including definition of venous thrombosis, anatomic location of thrombosis, and whether thromboses are symptomatic or clinically unsuspected to enhance comparisons across future studies. A better understanding of the epidemiology will be essential to inform further research to develop a risk stratification tool that facilitates early identification of neonates at high risk of venous thrombosis and effective strategies tailored to prevent thrombosis in NICU patients.

## Supplementary material

Appendix

PRISMA Checklist
