# Supplementary material for: Incidence of neonatal venous thromboembolism: a systematic review and meta-analysis of the literature
Source: Res Pract Thromb Haemost. 2026 May 8;10(4):106630. doi: 10.1016/j.rpth.2026.106630 (PMC13254879; doi:10.1016/j.rpth.2026.106630)
Supplement: Appendix [file mmc1.docx]

**Supplementary Material**

|  | **Description** | **Page** |
| --- | --- | --- |
| 1 | Supplementary Table S1. Search strategy used in Medline | 2 |
| 2 | Supplementary Table S2. Risk of bias in included studies | 3 |

**Supplementary Table S1. Search strategy used in Medline**

| **Concepts** | **Research strategy keywords** | **# Research** |
| --- | --- | --- |
| **Neonates** |  |  |
| Controlled vocabulary | exp infant/ or intensive care units, neonatal/ | 1 |
| Free text | (neonate* or neonatal or infant* or preterm or newborn* or NICU or NICUS or baby or babies or perinat* or PICU).ti,ab. | 2 |
|  | **#1 or #2** | 3 |
| **Thrombosis** |  |  |
|  | thrombosis/ or exp venous thrombosis/ or thromboembolism/ or venous thromboembolism/ | 4 |
|  | (thrombos* or thrombus or thrombi or phlebothrombo* or phlebo thrombo* or (blood adj3 clot*) or venothrombo* or thromboembol* or thrombo embol* or DVT or VTE).ti,ab. | 5 |
|  | **#4 OR #5** | 6 |
| **Risk factors** |  |  |
| Controlled vocabulary | exp risk/ or causality/ or precipitating factors/ or incidence/ or epidemiologic studies/ or exp cohort studies/ | 7 |
| Free text | (risk* or factor* or incidence* or predict* or association* or correlat* or relation* or interrelation* or likely or likel#hood* or odds or probab* or determinant* or cause* or causal* or causation* or etiolog* or aetiol* or link* or trigger* or genesis or connect* or epidemiol* or retrospect* or prospect* or ((cohort* or concurrent) adj2 stud*) or follow-up or followup or longitudinal).ti,ab. | 8 |
|  | **#7 or #8** | 9 |
|  | **#3 and #6 and #9** | 10 |
|  | **limit 10 to yr="1990 -Current"** | 11 |
|  | congress.pt. or congresses as topic/ or letter.pt. or editorial.pt. or comment.pt. or "consensus development conference, NIH".pt. or "consensus development conferences, NIH as topic"/ or consensus development conferences as topic/ or "consensus development conference".pt. or case reports.pt. | 12 |
|  | (congress* or letter or letters or editorial* or comment or comments or commentary or viewpoint or consensus development conference* or (case adj2 (report* or studies))).ti,ab. | 13 |
|  | **#12 or #13** | 14 |
|  | **#11 not #14** | 15 |

**Supplementary Table S2. Risk of bias in included studies**

| **Reference** | **Free of confounding** | **Free of selection bias** | **Adequate classification of exposure** | **No deviation from intended procedures** | **Adequate outcome data** | **Free of bias in outcome measurement** | **Free of selective reporting** |
| --- | --- | --- | --- | --- | --- | --- | --- |
| Aiyagari, 2012^1^ | No | Yes | Yes | Yes | Yes | Yes | Yes |
| Alten, 2012^2^ | No | Yes | Yes | Yes | Yes | Yes | Yes |
| Amankwah, 2014^3^ | Yes | Yes | Yes | Yes | Yes | No | Yes |
| Badur, 2021^4^ | No | Yes | Yes | Yes | Yes | Yes | Yes |
| Bhat, 2018^5^ | No | Yes | Yes | Yes | Yes | No | Yes |
| Bhat, 2022^6^ | No | Yes | Yes | Yes | Yes | No | Yes |
| Bhatia, 2022^7^ | No | No | Yes | Yes | Yes | No | No |
| Bokenkamp, 2000^8^ | No | No | Yes | Yes | Yes | No | Yes |
| Butler-O’Hara, 2006^9^ | Yes | Yes | Yes | Yes | Yes | Yes | Yes |
| Cabannes, 2018^10^ | Yes | Yes | Yes | Yes | Yes | No | Yes |
| Cakir, 2020^11^ | No | No | Yes | Yes | Yes | No | Yes |
| Chittihavorn, 2017^12^ | No | Yes | Yes | Yes | Yes | No | Unclear |
| Chojnacka, 2022^13^ | No | Yes | Unclear | Yes | Yes | No | Yes |
| Cholette, 2007^14^ | No | Yes | Yes | Yes | Yes | No | No |
| Claessens, 2018^15^ | No | Unclear | Yes | Yes | Yes | No | Yes |
| D’Andrea, 2023^16^ | Yes | Yes | Yes | Yes | Yes | Yes | Yes |
| Derinkuyu, 2017^17^ | Yes | Yes | Yes | Yes | Yes | Yes | Yes |
| Dubbink-Verheij, 2018^18^ | Yes | No | Yes | Yes | Yes | Yes | Yes |
| Dubbink-Verheij, 2020^19^ | Unclear | No | Yes | Yes | Yes | Yes | Unclear |
| Eason, 2020^20^ | Unclear | Yes | Unclear | Yes | Yes | Yes | Yes |
| Easterlin, 2022^21^ | Yes | Yes | No | Yes | Yes | No | Yes |
| El-Naggar, 2020^22^ | Yes | Unclear | Yes | Yes | Yes | Unclear | No |
| Faunes Perez, 2021^23^ | Yes | Unclear | Yes | Yes | Yes | Unclear | Yes |
| Gharehbaghi, 2011^24^ | No | Yes | Yes | Yes | Yes | Yes | Yes |
| Gibson, 2024^25^ | No | Yes | Yes | Yes | Yes | Unclear | Yes |
| Goh, 2021^26^ | No | Yes | Yes | Yes | Yes | Unclear | Yes |
| Guzoglu, 2023^27^ | No | Yes | No | Yes | Yes | No | Yes |
| Haddad, 2014^28^ | No | Yes | Yes | Unclear | Yes | Yes | Yes |
| Harrar, 2022^29^ | No | Yes | Yes | Yes | Yes | Yes | Yes |
| Haumont, 2008^30^ | Yes | Yes | Yes | Yes | Yes | Yes | Yes |
| Hess, 2023^31^ | No | No | Yes | Yes | No | No | No |
| Huerta, 2023^32^ | No | Yes | Yes | Yes | Unclear | Unclear | Yes |
| Hundsdoerfer, 2003^33^ | No | No | Yes | Yes | Yes | No | Yes |
| Hwang, 2020^34^ | Yes | Yes | Yes | Yes | Yes | No | Yes |
| Kim, 2001^35^ | Yes | No | Yes | Yes | Yes | No | Yes |
| Kisa, 2015^36^ | No | Yes | Yes | Yes | Yes | No | Yes |
| Lambert, 2019^37^ | No | Yes | Yes | Yes | Yes | No | Yes |
| Levit, 2020^38^ | No | Yes | Yes | Yes | Yes | No | Yes |
| Ma, 2015^39^ | No | Yes | Yes | Yes | Yes | No | Yes |
| Mehta, 1992^40^ | No | Yes | Unclear | Yes | Yes | Yes | Yes |
| Mirle, 2022^41^ | Unclear | Yes | Yes | Yes | Unclear | Yes | Yes |
| Murai, 2002^42^ | Yes | Yes | Yes | Yes | Yes | Yes | Yes |
| Narang, 2009^43^ | No | Yes | Yes | Yes | Yes | Yes | No |
| Navaratnam, 2023^44^ | No | Yes | Yes | Yes | Yes | No | Yes |
| Nemati, 2013^45^ | No | Yes | Yes | Yes | Yes | Yes | Yes |
| Ouellette, 2020^46^ | No | Yes | Yes | Yes | Yes | Unclear | Yes |
| Paes, 2022^47^ | No | Yes | Yes | Yes | Yes | Yes | Yes |
| Patregnani, 2018^48^ | No | Yes | Yes | Yes | Yes | No | Yes |
| Perez-Perez, 2023^49^ | No | Unclear | Unclear | Yes | Yes | Yes | Yes |
| Pippus, 1994^50^ | No | Yes | Yes | Yes | Yes | Yes | Yes |
| Puetz, 2009^51^ | No | No | Yes | Unclear | Yes | No | Yes |
| Raets, 2013^52^ | No | Yes | Yes | Yes | Yes | Yes | No |
| Ratchagame, 2021^53^ | No | Unclear | Yes | Yes | Yes | Unclear | Yes |
| Roberts, 1990^54^ | No | Yes | Yes | Yes | Yes | Yes | Yes |
| Rohr, 2014^55^ | No | Yes | Yes | Unclear | Yes | Yes | Yes |
| Roy, 2002^56^ | Yes | Yes | Yes | Yes | No | Yes | Yes |
| Rubio Longo, 2021^57^ | No | Yes | Yes | Yes | Yes | Yes | No |
| Sakha, 2007^58^ | No | No | Yes | Yes | Yes | Unclear | No |
| Salonvaara, 1999^59^ | No | No | Yes | Yes | Yes | Yes | Yes |
| Schmidt, 1995^60^ | Yes | Yes | Yes | Yes | No | Yes | Yes |
| Schwartz, 1997^61^ | Yes | Yes | Yes | Yes | Unclear | Yes | Yes |
| Shah, 2015^62^ | Yes | Yes | Yes | Yes | Yes | Unclear | Yes |
| Shah, 2007^63^ | Yes | Yes | Yes | Yes | Unclear | Yes | Yes |
| Shalaby, 2021^64^ | No | No | Yes | Yes | Yes | Yes | Yes |
| Sirachainan, 2018 ^65^ | No | Yes | Yes | Yes | Yes | Yes | Yes |
| Sobczak, 2021^66^ | No | Yes | Yes | Yes | Yes | Yes | Yes |
| Sobczak, 2024^67^ | No | Yes | Yes | Yes | Yes | Yes | Yes |
| Sorg, 2021^68^ | No | Unclear | Yes | Yes | Yes | Yes | Yes |
| Stein, 2019^69^ | Yes | Yes | Yes | Yes | Yes | Yes | Yes |
| Stewart, 2022^70^ | Yes | Yes | Yes | Yes | Yes | Yes | Yes |
| Swartz, 2022^71^ | No | No | Yes | Yes | Yes | Yes | Yes |
| Tewary, 2024^72^ | No | Yes | Yes | Yes | Unclear | Unclear | Yes |
| Thornburg, 2007^73^ | No | Yes | Yes | Yes | Yes | Yes | Yes |
| Tsai, 2011^74^ | No | Yes | Yes | Yes | Yes | Yes | No |
| Tuckuviene, 2012^75^ | No | Yes | No | Yes | Yes | Yes | Yes |
| Turebylu, 2007^76^ | Yes | Yes | Yes | Yes | Yes | Yes | Yes |
| Ulloa-Ricardez, 2015^77^ | No | No | Yes | Yes | Yes | No | Yes |
| Unal, 2012^78^ | Yes | Yes | Yes | Yes | Yes | Yes | Yes |
| Uslu, 2010^79^ | Yes | Yes | Yes | Yes | Yes | Yes | Yes |
| van Ommen, 2023^80^ | No | No | Yes | Yes | Yes | No | Yes |
| White, 2020^81^ | Yes | Yes | Yes | Yes | Yes | No | Yes |
| Zhu, 2022^82^ | No | Yes | Yes | Yes | Yes | Yes | No |

**REFERENCES**

Regression Test for Asymmetry: Z=7.695, p<0.001

1. Aiyagari R, Song JY, Donohue JE, Yu S, Gaies MG. Central venous catheter-associated complications in infants with single ventricle: comparison of umbilical and femoral venous access routes. *Pediatric Critical Care Medicine*. 2012;13(5):549-553. doi:<https://dx.doi.org/10.1097/PCC.0b013e31824fbdb4>

2. Alten JA, Borasino S, Gurley WQ, Law MA, Toms R, Dabal RJ. Ultrasound-guided femoral vein catheterization in neonates with cardiac disease. *Pediatric Critical Care Medicine*. 2012;13(6):654-659. doi:<https://dx.doi.org/10.1097/PCC.0b013e318250af0c>

3. Amankwah EK, Atchison CM, Arlikar S, et al. Risk factors for hospital-sssociated venous thromboembolism in the neonatal intensive care unit. *Thrombosis Research*. 2014;134(2):305-309. doi:<https://dx.doi.org/10.1016/j.thromres.2014.05.036>

4. Badur CA, Buhrer C, Dame C. Adult Donor Blood Products as Risk Factors for Central Venous Catheter-associated Thromboembolism in Neonates: A Retrospective Case-Control Study. *Journal of Pediatric Hematology/Oncology*. 2021;43(2):e255-e259. doi:<https://dx.doi.org/10.1097/MPH.0000000000001821>

5. Bhat R, Kumar R, Kwon S, Murthy K, Liem RI. Risk Factors for Neonatal Venous and Arterial Thromboembolism in the Neonatal Intensive Care Unit-A Case Control Study. *Journal of Pediatrics*. 2018;195:28-32. doi:<https://dx.doi.org/10.1016/j.jpeds.2017.12.015>

6. Bhat R, Kwon S, Zaniletti I, Murthy K, Liem RI. Risk factors associated with venous and arterial neonatal thrombosis in the intensive care unit: a multicentre case-control study. *The Lancet Haematology*. 2022;9(3):e200-e207. doi:<https://dx.doi.org/10.1016/S2352-3026(21)00399-9>

7. Bhatia K, Solanki S, Paes B, Chan AKC, Bhatt MD. Risk factors for neonatal thrombosis: A retrospective study conducted in a single Canadian intensive care unit. *Pediatric Blood & Cancer*. 2022;69(6):e29668. doi:<https://dx.doi.org/10.1002/pbc.29668>

8. Bokencamp A, Von Kries R, Nowak-Gottl U, Göbel U, Hoyer PF. Neonatal renal venous thrombosis in Germany between 1992 and 1994: epidemiology, treatment and outcome. *Eur J Pediatr*. 2000;doi:10.1016/j.athoracsur.2013.04.061

9. Butler-O'Hara M, Buzzard CJ, Reubens L, McDermott MP, DiGrazio W, D'Angio CT. A randomized trial comparing long-term and short-term use of umbilical venous catheters in premature infants with birth weights of less than 1251 grams. *Pediatrics*. 2006;118(1):e25-35.

10. Cabannes M, Bouissou A, Favrais G, et al. Systematic ultrasound examinations in neonates admitted to NICU: evolution of portal vein thrombosis. *Journal of Perinatology*. 2018;38(10):1359-1364. doi:<https://dx.doi.org/10.1038/s41372-018-0132-9>

11. Cakir SC, Ozkan H, Dorum BA, et al. The danger awaiting premature babies: Portal vein thrombosis. *Turk Pediatri Arsivi*. 2020;55(3):257-262. doi:<https://dx.doi.org/10.14744/TurkPediatriArs.2020.65289>

12. Chittithavorn V, Duangpakdee P, Rergkliang C, Pruekprasert N. Risk factors for in-hospital shunt thrombosis and mortality in patients weighing less than 3 kg with functionally univentricular heart undergoing a modified Blalock-Taussig shunt. *Interactive Cardiovascular & Thoracic Surgery*. 2017;25(3):407-413. doi:<https://dx.doi.org/10.1093/icvts/ivx147>

13. Chojnacka K, Krasinski Z, Wroblewska-Seniuk K, Mazela J. Catheter-related venous thrombosis in NICU: A case-control retrospective study. *Journal of Vascular Access*. 2022;23(1):88-93. doi:<https://dx.doi.org/10.1177/1129729820983203>

14. Cholette JM, Rubenstein JS, Alfieris GM, et al. Elevated risk of thrombosis in neonates undergoing initial palliative cardiac surgery. *Annals of Thoracic Surgery*. 2007;84(4):1320-1325.

15. Claessens NHP, Algra SO, Jansen NJG, et al. Clinical and neuroimaging characteristics of cerebral sinovenous thrombosis in neonates undergoing cardiac surgery. *Journal of Thoracic & Cardiovascular Surgery*. 2018;155(3):1150-1158. doi:<https://dx.doi.org/10.1016/j.jtcvs.2017.10.083>

16. D'Andrea V, Prontera G, Pinna G, et al. Securement of Umbilical Venous Catheter Using Cyanoacrylate Glue: A Randomized Controlled Trial. *The Journal of pediatrics*. 2023;260:113517. doi:<https://dx.doi.org/10.1016/j.jpeds.2023.113517>

17. Derinkuyu BE, Boyunaga OL, Damar C, et al. Hepatic Complications of Umbilical Venous Catheters in the Neonatal Period: The Ultrasound Spectrum. *Journal of Ultrasound in Medicine*. 2018;37(6):1335-1344. doi:<https://dx.doi.org/10.1002/jum.14443>

18. Dubbink-Verheij GH, Pelsma ICM, van Ommen CH, et al. Femoral Vein Catheter is an Important Risk Factor for Catheter-related Thrombosis in (Near-)term Neonates. *Journal of Pediatric Hematology/Oncology*. 2018;40(2):e64-e68. doi:<https://dx.doi.org/10.1097/MPH.0000000000000978>

19. Dubbink-Verheij GH, Visser R, Roest AA, van Ommen CH, Te Pas AB, Lopriore E. Thrombosis after umbilical venous catheterisation: prospective study with serial ultrasound. *Archives of Disease in Childhood Fetal & Neonatal Edition*. 2020;105(3):299-303. doi:<https://dx.doi.org/10.1136/archdischild-2018-316762>

20. Eason AJ, Crethers D, Ghosh S, Stansfield BK, Polimenakos AC. Central Vascular Thrombosis in Neonates with Congenital Heart Disease Awaiting Cardiac Intervention. *Pediatric Cardiology*. 2020;41(7):1340-1345. doi:<https://dx.doi.org/10.1007/s00246-020-02383-2>

21. Easterlin MC, Li Y, Yieh L, et al. Predictors of venous thromboembolism among infants in children's hospitals in the United States: a retrospective Pediatric Health Information Study. *Journal of Perinatology*. 2022;42(1):103-109. doi:<https://dx.doi.org/10.1038/s41372-021-01232-1>

22. El-Naggar W, Yoon EW, McMillan D, et al. Epidemiology of thrombosis in Canadian neonatal intensive care units. *Journal of Perinatology*. 2020;40(7):1083-1090. doi:<https://dx.doi.org/10.1038/s41372-020-0678-1>

23. Faunes Perez M, Gonzalez Morande A, Perez Arriaran ME, et al. Risk factors associated with complications of peripherally inserted central catheter in newborn infants. *Andes Pediatrica : Revista Chilena de Pediatria*. 2021;92(5):710-717. doi:<https://dx.doi.org/10.32641/andespediatr.v92i5.3526>

24. Gharehbaghi MM, Nemati M, Hosseinpour SS, Taei R, Ghargharechi R. Umbilical vascular catheter associated portal vein thrombosis detected by ultrasound. *Indian Journal of Pediatrics*. 2011;78(2):161-164. doi:<https://dx.doi.org/10.1007/s12098-010-0223-x>

25. Gibson K, Smith A, Sharp R, Ullman A, Morris S, Esterman A. Adverse events associated with umbilical vascular catheters in the neonatal intensive care unit: A retrospective cohort study. *Australian critical care : official journal of the Confederation of Australian Critical Care Nurses*. 2024;doi:<https://dx.doi.org/10.1016/j.aucc.2024.01.013>

26. Goh SSM, Kan SY, Bharadwaj S, Poon WB. A review of umbilical venous catheter-related complications at a tertiary neonatal unit in Singapore. *Singapore Medical Journal*. 2021;62(1):29-33. doi:<https://dx.doi.org/10.11622/smedj.2019140>

27. Guzoglu N, Albayrak M, Aliefendioglu D. Evaluation of Patients with Neonatal Thrombosis. *Indian journal of pediatrics*. 2023;90(6):615-617. doi:<https://dx.doi.org/10.1007/s12098-023-04497-w>

28. Haddad H, Lee KS, Higgins A, McMillan D, Price V, El-Naggar W. Routine surveillance ultrasound for the management of central venous catheters in neonates. *Journal of Pediatrics*. 2014;164(1):118-122. doi:<https://dx.doi.org/10.1016/j.jpeds.2013.08.048>

29. Harrar DB, Goss M, Donofrio MT, et al. Cerebral Sinus Venous Thrombosis in Infants after Surgery for Congenital Heart Disease. *Journal of Pediatrics*. 2022;248:59-65.e53. doi:<https://dx.doi.org/10.1016/j.jpeds.2022.05.056>

30. Haumont D, de Beauregard VG, Van Herreweghe I, Delanghe G, Ciardelli R, Haelterman E. A new technique for transumbilical insertion of central venous silicone catheters in newborn infants. *Acta Paediatrica*. 2008;97(7):988-990. doi:<https://dx.doi.org/10.1111/j.1651-2227.2008.00786.x>

31. Hess S, Poryo M, Bottger R, et al. Umbilical venous catheter- and peripherally inserted central catheter-associated complications in preterm infants with birth weight <1250 g : Results from a survey in Austria and Germany. *Nabelvenenkatheter- und periphere zentrale katheterassoziierte Komplikationen bei Fruhgeborenen mit einem Geburtsgewicht <1250 g : Ergebnisse einer Umfrage in Osterreich und Deutschland*. 2023;173(7-8):161-167. doi:<https://dx.doi.org/10.1007/s10354-022-00952-z>

32. Huerta CT, Saberi RA, Lynn R, et al. Outcomes After Ladd Procedures for Intestinal Malrotation in Newborns with Heterotaxy Syndrome. *Journal of pediatric surgery*. 2023;58(6):1095-1100. doi:<https://dx.doi.org/10.1016/j.jpedsurg.2023.02.013>

33. Hundsdoerfer P, Vetter B, Stover B, et al. Homozygous and double heterozygous Factor V Leiden and Factor II G20210A genotypes predispose infants to thromboembolism but are not associated with an increase of foetal loss. *Thrombosis and Haemostasis*. 2003;90(4):628-635. doi:<https://dx.doi.org/10.1160/th03-02-0096>

34. Hwang JH, Chung ML, Lim YJ. Incidence and risk factors of subclinical umbilical catheter-related thrombosis in neonates. *Thrombosis Research*. 2020;194:21-25. doi:<https://dx.doi.org/10.1016/j.thromres.2020.05.034>

35. Kim JH, Lee YS, Kim SH, Lee SK, Lim MK, Kim HS. Does umbilical vein catheterization lead to portal venous thrombosis? Prospective US evaluation in 100 neonates. *Radiology*. 2001;219(3):645-650.

36. Kisa P, Ting J, Callejas A, Osiovich H, Butterworth SA. Major thrombotic complications with lower limb PICCs in surgical neonates. *Journal of Pediatric Surgery*. 2015;50(5):786-789. doi:<https://dx.doi.org/10.1016/j.jpedsurg.2015.02.043>

37. Lambert I, Tarima S, Uhing M, Cohen SS. Risk Factors Linked to Central Catheter-Associated Thrombosis in Critically Ill Infants in the Neonatal Intensive Care Unit. *American Journal of Perinatology*. 2019;36(3):291-295. doi:<https://dx.doi.org/10.1055/s-0038-1667377>

38. Levit OL, Shabanova V, Bizzarro MJ. Umbilical catheter-associated complications in a level IV neonatal intensive care unit. *Journal of Perinatology*. 2020;40(4):573-580. doi:<https://dx.doi.org/10.1038/s41372-019-0579-3>

39. Ma M, Garingo A, Jensen AR, Bliss D, Friedlich P. Complication risks associated with lower versus upper extremity peripherally inserted central venous catheters in neonates with gastroschisis. *Journal of Pediatric Surgery*. 2015;50(4):556-558. doi:<https://dx.doi.org/10.1016/j.jpedsurg.2014.08.026>

40. Mehta S, Connors AF, Jr., Danish EH, Grisoni E. Incidence of thrombosis during central venous catheterization of newborns: a prospective study. *Journal of Pediatric Surgery*. 1992;27(1):18-22.

41. Mirle N, Rajan A, Shriyan A, Soans S. Quality Improvement Initiative for Neonates: Use of In-line Endotoxin Filters in Central Venous Catheters: A Prospective Interventional Study. *Journal of Clinical and Diagnostic Research*. 2022;16(11):SC09-SC12. doi:<https://dx.doi.org/10.7860/JCDR/2022/59412.17181>

42. Murai DT. Are femoral Broviac catheters effective and safe? A prospective comparison of femoral and jugular venous broviac catheters in newborn infants. *Chest*. 2002;121(5):1527-1530.

43. Narang S, Roy J, Stevens TP, Butler-O'Hara M, Mullen CA, D'Angio CT. Risk factors for umbilical venous catheter-associated thrombosis in very low birth weight infants. *Pediatric Blood & Cancer*. 2009;52(1):75-79. doi:<https://dx.doi.org/10.1002/pbc.21714>

44. Navaratnam M, Mendoza JM, Zhang S, et al. Activated 4-Factor Prothrombin Complex Concentrate as a Hemostatic Adjunct for Neonatal Cardiac Surgery: A Propensity Score-Matched Cohort Study. *Anesthesia and analgesia*. 2023;136(3):473-482. doi:<https://dx.doi.org/10.1213/ANE.0000000000006294>

45. Nemati M, Gharehbaghi MM, Shakeri A, Nobari RT, Behravan N, Goldust M. Vein thrombosis associated with umbilical vascular catheters with color doppler. *Journal of Biological Sciences*. 2013;13(8):722-726. doi:<https://dx.doi.org/10.3923/jbs.2013.722.726>

46. Ouellette AC, Darling EK, Sivapathasundaram B, et al. Incidence, Risk Factors, and Outcomes of Neonatal Renal Vein Thrombosis in Ontario: Population-Based Cohort Study. *Kidney360*. 2020;1(7):640-647. doi:<https://dx.doi.org/10.34067/KID.0000912019>

47. Paes B, Chan AKC, Shaik M, et al. Epidemiology, diagnosis and management of neonatal thrombosis: a single-center cohort study. *Blood Coagulation & Fibrinolysis*. 2022;33(2):83-89. doi:<https://dx.doi.org/10.1097/MBC.0000000000001110>

48. Patregnani JT, Sochet AA, Zurakowski D, et al. Cardiopulmonary Bypass Reduces Early Thrombosis of Systemic-to-Pulmonary Artery Shunts. *World Journal for Pediatric & Congenital Heart Surgery*. 2018;9(3):276-282. doi:<https://dx.doi.org/10.1177/2150135118755985>

49. Perez-Perez A, Vigil-Vazquez S, Gutierrez-Velez A, et al. Chylothorax in newborns after cardiac surgery: a rare complication? *European journal of pediatrics*. 2023;182(4):1569-1578. doi:<https://dx.doi.org/10.1007/s00431-023-04808-5>

50. Pippus KG, Giacomantonio JM, Gillis DA, Rees EP. Thrombotic complications of saphenous central venous lines. *Journal of Pediatric Surgery*. 1994;29(9):1218-1219.

51. Puetz J, Darling G, Brabec P, Blatny J, Mathew P. Thrombotic events in neonates receiving recombinant factor VIIa or fresh frozen plasma. *Pediatric Blood & Cancer*. 2009;53(6):1074-1078. doi:<https://dx.doi.org/10.1002/pbc.22160>

52. Raets MM, Sol JJ, Govaert P, et al. Serial cranial US for detection of cerebral sinovenous thrombosis in preterm infants. *Radiology*. 2013;269(3):879-886. doi:<https://dx.doi.org/10.1148/radiol.13130401>

53. Ratchagame V, Prabakaran V. Comparison of Risks from Central Venous Catheters and Peripheral Intravenous Lines among Term Neonates in a Tertiary Care Hospital, India. *Journal of Caring Sciences*. 2021;10(2):57-61. doi:<https://dx.doi.org/10.34172/jcs.2021.012>

54. Roberts JP, Gollow IJ. Central venous catheters in surgical neonates. *Journal of Pediatric Surgery*. 1990;25(6):632-634. doi:<https://dx.doi.org/10.1016/0022-3468%2890%2990350-I>

55. Rohr SB, Sauer H, Gottschling S, et al. Non-neurological, steroid-related adverse events in very low birth weight infants: a prospective audit. *Swiss Medical Weekly*. 2014;144:w13954. doi:<https://dx.doi.org/10.4414/smw.2014.13954>

56. Roy M, Turner-Gomes S, Gill G, Way C, Mernagh J, Schmidt B. Accuracy of Doppler echocardiography for the diagnosis of thrombosis associated with umbilical venous catheters. *Journal of Pediatrics*. 2002;140(1):131-134.

57. Rubio Longo MC, De Lucca PM, Goldsmit G, Farina D, Lipsich J, Rodriguez S. Catheter-related deep vein thrombosis in newborn infants. *Archivos Argentinos de Pediatria*. 2021;119(1):32-38. doi:<https://dx.doi.org/10.5546/aap.2021.eng.32>

58. Sakha SH, Rafeey M, Tarzamani MK. Portal venous thrombosis after umbilical vein catheterization. *Indian Journal of Gastroenterology*. 2007;26(6):283-284.

59. Salonvaara M, Riikonen P, Kekomaki R, Heinonen K. Clinically symptomatic central venous catheter-related deep venous thrombosis in newborns. *Acta Paediatrica*. 1999;88(6):642-646.

60. Schmidt B, Andrew M. Neonatal thrombosis: report of a prospective Canadian and international registry. *Pediatrics*. 1995;96(5 Pt 1):939-943.

61. Schwartz DS, Gettner PA, Konstantino MM, et al. Umbilical venous catheterization and the risk of portal vein thrombosis. *Journal of Pediatrics*. 1997;131(5):760-762.

62. Shah SH, West AN, Sepanski RJ, Hannah D, May WN, Anand KJ. Clinical risk factors for central line-associated venous thrombosis in children. *Frontiers in Pediatrics*. 2015;3:35. doi:<https://dx.doi.org/10.3389/fped.2015.00035>

63. Shah PS, Kalyn A, Satodia P, et al. A randomized, controlled trial of heparin versus placebo infusion to prolong the usability of peripherally placed percutaneous central venous catheters (PCVCs) in neonates: the HIP (Heparin Infusion for PCVC) study. *Pediatrics*. 2007;119(1):e284-291.

64. Shalaby MM, Salama RM, Mansour MA. Modified Seldinger technique for internal jugular open central venous line insertion in neonates: merging two different manoeuvers. *Egyptian Pediatric Association Gazette*. 2021;69(1):30. doi:<https://dx.doi.org/10.1186/s43054-021-00078-8>

65. Sirachainan N, Limrungsikul A, Chuansumrit A, et al. Incidences, risk factors and outcomes of neonatal thromboembolism. *Journal of Maternal-Fetal & Neonatal Medicine*. 2018;31(3):347-351. doi:<https://dx.doi.org/10.1080/14767058.2017.1285892>

66. Sobczak A, Dudzik A, Kruczek P, Kwinta P. Ultrasound Monitoring of Umbilical Catheters in the Neonatal Intensive Care Unit-A Prospective Observational Study. *Frontiers in Pediatrics*. 2021;9:665214. doi:<https://dx.doi.org/10.3389/fped.2021.665214>

67. Sobczak A, Kowalik A, Homa M, Turalska P, Kwinta P. Changes in umbilical catheters' microstructure in vivo: A prospective study. *The journal of vascular access*. 2024;25(1):158-164. doi:<https://dx.doi.org/10.1177/11297298221100441>

68. Sorg AL, Von Kries R, Klemme M, et al. Incidence and risk factors of cerebral sinovenous thrombosis in infants. *Developmental Medicine & Child Neurology*. 2021;63(6):697-704. doi:<https://dx.doi.org/10.1111/dmcn.14816>

69. Stein ML, Quinonez LG, DiNardo JA, Brown ML. Complications of Transthoracic Intracardiac and Central Venous Lines in Neonates Undergoing Cardiac Surgery. *Pediatric Cardiology*. 2019;40(4):733-737. doi:<https://dx.doi.org/10.1007/s00246-019-02057-8>

70. Stewart LA, Klein-Cloud R, Gerall C, et al. Extracorporeal Membrane Oxygenation (ECMO) and its complications in newborns with congenital diaphragmatic hernia. *Journal of Pediatric Surgery*. 2022;57(8):1642-1648. doi:<https://dx.doi.org/10.1016/j.jpedsurg.2021.12.028>

71. Swartz MF, Hutchinson DJ, Stauber SD, Taillie ER, Alfieris GM, Cholette JM. Enoxaparin Reduces Catheter-associated Venous Thrombosis After Infant Cardiac Surgery. *Annals of Thoracic Surgery*. 2022;114(3):881-888. doi:<https://dx.doi.org/10.1016/j.athoracsur.2021.05.009>

72. Tewary S, Sontakke S, Dean K, Ellis D, Ghose A, Kanthimathinathan HK. Examining safety and efficacy of a fixed concentration heparin dosing strategy for anticoagulation in neonatal extracorporeal membrane oxygenation. *Perfusion (United Kingdom)*. 2024;39(5):869-875. doi:<https://dx.doi.org/10.1177/02676591231167709>

73. Thornburg CD, Smith PB, Smithwick ML, Cotten CM, Benjamin DK, Jr. Association between thrombosis and bloodstream infection in neonates with peripherally inserted catheters. *Thrombosis Research*. 2008;122(6):782-785.

74. Tsai MH, Chu SM, Lien R, et al. Complications associated with 2 different types of percutaneously inserted central venous catheters in very low birth weight infants. *Infection Control and Hospital Epidemiology*. 2011;32(3):258-266. doi:<https://dx.doi.org/10.1086/658335>

75. Tuckuviene R, Christensen AL, Helgested J, Hundborg HH, Kristensen SR, Johnsen SP. Infant, obstetrical and maternal characteristics associated with thromboembolism in infancy: a nationwide population-based case-control study. *Archives of Disease in Childhood Fetal & Neonatal Edition*. 2012;97(6):F417-422. doi:<https://dx.doi.org/10.1136/archdischild-2011-300665>

76. Turebylu R, Salis R, Erbe R, Martin D, Lakshminrusimha S, Ryan RM. Genetic prothrombotic mutations are common in neonates but are not associated with umbilical catheter-associated thrombosis. *Journal of Perinatology*. 2007;27(8):490-495.

77. Ulloa-Ricardez A, Romero-Espinoza L, Estrada-Loza Mde J, Gonzalez-Cabello HJ, Nunez-Enriquez JC. Risk Factors for Intracardiac Thrombosis in the Right Atrium and Superior Vena Cava in Critically Ill Neonates who Required the Installation of a Central Venous Catheter. *Pediatrics & Neonatology*. 2016;57(4):288-294. doi:<https://dx.doi.org/10.1016/j.pedneo.2015.10.001>

78. Unal S, Ekici F, Cetin, II, Bilgin L. Heparin infusion to prevent umbilical venous catheter related thrombosis in neonates. *Thrombosis Research*. 2012;130(5):725-728. doi:<https://dx.doi.org/10.1016/j.thromres.2012.07.018>

79. Uslu S, Ozdemir H, Comert S, Bolat F, Nuhoglu A. The effect of low-dose heparin on maintaining peripherally inserted percutaneous central venous catheters in neonates. *Journal of Perinatology*. 2010;30(12):794-799. doi:<https://dx.doi.org/10.1038/jp.2010.46>

80. van Ommen CH, Bergman KA, Boerma M, et al. NEOnatal Central-venous Line Observational study on Thrombosis (NEOCLOT): evaluation of a national guideline on management of neonatal catheter-related venous thrombosis. *Journal of thrombosis and haemostasis : JTH*. 2023;21(4):963-974. doi:<https://dx.doi.org/10.1016/j.jtha.2022.11.044>

81. White MH, Kelleman M, Sidonio RF, Jr., Kochilas L, Patel KN. Incidence and Timing of Thrombosis After the Norwood Procedure in the Single-Ventricle Reconstruction Trial. *Journal of the American Heart Association*. 2020;9(24):e015882. doi:<https://dx.doi.org/10.1161/JAHA.120.015882>

82. Zhu W, Zhang H, Xing Y. Clinical Characteristics of Venous Thrombosis Associated with Peripherally Inserted Central Venous Catheter in Premature Infants. *Children*. 2022;9(8):28. doi:<https://dx.doi.org/10.3390/children9081126>
